# Conscious Sedation with Nitrous Oxide to control Stress during Dental Treatment in Patients with Cerebral Palsy: An Experimental Clinical Trial

**DOI:** 10.5005/jp-journals-10005-1470

**Published:** 2017-02-27

**Authors:** Fernando M Baeder, Daniel F Silva, Ana CL de Albuquerque, Maria TBR Santos

**Affiliations:** 1 Professor, Department of Dentistry, Universidade Cruzeiro do Sul, Sao Paulo, Brazil; 2 Professor, Department of Dentistry, Faculdades Integradas de Patos Paraiba, Brazil; 3 Professor, Department of Dentistry, Universidade Federal de Campina Grande, Paraiba, Brazil; 4 Professor, Department of Dentistry, Universidade Cruzeiro do Sul, Sao Paulo, Brazil

**Keywords:** Cerebral palsy, Clinical trial, Conscious sedation, Dental care, Nitrous oxide.

## Abstract

**Introduction:**

Individuals with cerebral palsy (CP) often present with oral alterations that impact oral health and require dental treatment.

**Aim:**

This study aimed to evaluate the use of conscious sedation with nitrous oxide (N_2_O) to control stress during dental treatment in individuals with CP using as parameters: Venham score (VS), heart rate (HR), and respiratory rate (RR).

**Materials and methods:**

A total of 77 CP patients >3 years of age with a mean age of 11.8 (± 6.4) years were evaluated in a rehabilitation center. Stress control was measured at the following time points: T1 (presedation), T2 (induction), T3 (sedated patient), and T4 (end). Student’s t-test, the Chi-squared test, analysis of variance (ANOVA), and the McNemar test were used. The significance level was 5%.

**Results:**

Sedation ranged between 10 and 60% N_2_O, with an average of 35.6% (± 10.4). The RR did not vary among the times (p = 0.12). The HR and VS varied significantly between times (p < 0.001), as significantly higher values of HR were observed at T1.

**Conclusion:**

Conscious sedation with N_2_O during dental care controls stress in CP patients, as verified by a decrease in HR, and does not promote respiratory depression. Higher concentrations of N_2_O are recommended for CP patients with tachycardia.

**Clinical significance:**

Sedation modifies behavior during dental procedures, facilitating patient collaboration.

**How to cite this article:** Baeder FM, Silva DF, de Albuquerque ACL, Santos MTBR. Conscious Sedation with Nitrous Oxide to control Stress during Dental Treatment in Patients with Cerebral Palsy: An Experimental Clinical Trial. Int J Clin Pediatr Dent 2017;10(4):384-390.

## INTRODUCTION

Cerebral palsy is a disabling condition related to neuro-development. The CP causes severe motor impairment, which when coupled with associated conditions, such as intellectual disability results in reduced intraoral self-cleaning function. The CP generates abnormal involuntary movements of facial and mastication-associated muscles, as well as oral pathological reflexes that negatively impact oral health. In some cases, upper limb involvement may complicate the movements required for brushing, thus facilitating biofilm accumulation and fostering the increased prevalence of oral diseases, heightening the need for dental care.^1-5^

The lack of cooperation by patients with CP during dental procedures may originate from the physical discomfort caused by situations inducing fear and anxiety.^6^ Therefore, dental procedures are often performed under general anesthesia in these patients.^7^ However, from both a physiological and a behavioral perspective, conscious sedation with N_2_O may be an alternative resource for patients who prefer dental care in an outpatient setting.^8,9^ Due to the lack of studies in the literature regarding the use of conscious sedation with N_2_O for dental care in individuals with CP, the objective of this study was to evaluate its use with respect to stress control during dental care in individuals with CP, using the following physiological and behavioral parameters: HR, RR, and VS.

## MATERIALS AND METHODS

This study was a nonrandomized experimental trial conducted between 2011 and 2012. The sampling method was performed by convenience. The patients were invited and agreed to participate in this study at the time of data collection. The study included 77 patients with CP, who attended the outpatient clinic of Lar Escola Sao Francisco. No patient provided reasons for exclusion from the sample.

The inclusion criteria included patients with a medical diagnosis of CP, patients older than 3 years of age, patients with any clinical patterns suggestive of CP, patients with caries in at least two molars (primary or permanent), and patients with an indication for the use of an anesthetic to perform restorative procedures. The exclusion criteria included patients with chronic respiratory problems, such as chronic bronchitis and chronic obstructive pulmonary disease, as well as acute respiratory conditions, such as influenza with nasal congestion and active pneumonia, as these conditions are contraindications to the use of N_2_O; patients with CP with associated genetic syndromes, as well as patients whose caregivers refused to provide informed consent, were excluded. Prior to data collection, this study was approved under protocol CE/UCS—044/2010 and clinical trial number NCT02322983.

Physiological parameters, such as HR and RR were measured by a professional using an Ianum Monitor, LCD TFT 8.4®, which was manufactured in the USA for adult, pediatric, and neonatal use. For analgesia, the MATRX flowmeter was used (an MDM model under the ANVISA registration No. 900507301215). The sedation equipment contained a flowmeter, which was responsible for mixing and titrating the gases (O_2_/N_2_O). These titrations may vary from 10 to 70% and are controlled by a single professional, who establishes the percentages depending on the required level of clinical sedation. Two dentists assessed the patients’ behavior using the VS, one of whom was unaware of the type of sedation used.

Dental care routines were established by standardizing the procedures and ensuring the reproducibility of the sedation method. During the initial consultation, a medical history was obtained (sociodemographic data of the participants), and a dental examination was performed. Data relating to the clinical standards for CP were collected from the patients’ medical records and were noted in the evaluation of the dental records described herein. Clarification regarding both the sedation technique and treatment plan was provided to the patients’ guardians.

During the dental examination, caries were diagnosed in accordance with the diagnostic criteria proposed by the World Health Organization.^10^ During the second visit, the adaptation and selection of an appropriate nasal mask were undertaken, and the monitoring equipment was positioned, followed by observations and recordings of the patients’ behavioral and physiological records using evaluation sheets. The relative proportions of N_2_O/O_2_ were manipulated to achieve optimal sedation.^11-14^ In the present study, good sedation of a patient with CP entailed achieving a state of relaxation sufficient to facilitate improved cooperation with treatment.

Behavior during dental treatment was evaluated using the VS,^15^ which is used for behavioral assessments of dental patients. The assessment was performed based on the behavior of the patient during the dental procedure. The VS values were determined based on the following definitions:

- *Relaxed:* Fully cooperative, smiling, helpful, willing to talk;
- *Apprehensive:* Worried, claims slight discomfort, keeps hands lowered or partially raised, and expresses facial tension but is able to cooperate;
- *Tense:* Reflects anxiety by tone of voice during questioning, exhibits stress during the procedure, as well as verbal protests, crying, raised hands, and a tense disposition, but does not interfere very much and protests to distract and disturb;
- *Reluctant:* Exhibits energetic verbal protesting, crying, and use of the hands to stop the procedure, but the procedure continues with difficulty;
- *Interruption:* Exhibits continual weeping with body movements sometimes requiring mechanical restraint; the protests interrupt the procedure;
- *Without communication:* Cries very loudly, sweats, screams, is unable to hear, attempts to escape; these patients require mechanical restraints.

Behavioral and physiological parameter evaluations for the patients referred for dental treatment were performed in four stages, as follows:

1. *T1 (presedation):* the patient’s baseline physiological parameters and behavior were assessed using VSs at the moment the patient was seated in the chair;
2. *T2 (induction):* measured 5 minutes after placing the mask and achieving the optimal titration with which to sedate the patient;
3. *T3 (sedated patient):* measured 10 minutes after initiating the restorative procedure in the superior first molar (deciduous or permanent); all of the patients in this phase received dental treatment under local anesthesia with mepivacaine (epinephrine 1:100,000), using a dosage based on weight;^16^
4. *T4 (end):* 5 minutes following the removal of N_2_O (oxygenation maintenance).

The VS, HR, and RR were measured at each of the time intervals. The VS was later associated with cooperation, defining the patient as either a collaborator or a noncollaborator. The HR was later corrected for physiological cardiac variations among the different age groups. Each of the patients exhibited normal cardiac function^17^ (Table 1).

**Table 1:** **Table 1:** Heart rate variability in different age groups

*Age*		*Beats per minute*	
<1 year		110-130	
1-3 years		90-115	
4-14 years		80-105	
14-21 years		78-85	
Over 21 years		60-75	

Continuous variables were compared between two groups using Student’s t-test, and factors were compared among three or more groups via ANOVA.^18,19^ The McNemar test was used to evaluate behavior according to the patients’ VSs.^20^ In order evaluate the variables (HR, RR, and VS) over time, nonparametric models were adjusted for repeated measures using Brunner et al test.^21^ The calculations were performed using R 3.0.2 statistical software (R Core Team, 2014). A significance level of 5% was established. The graphs were generated using *ggplot2.^22^*

## RESULTS

The results are presented as relative and absolute frequencies for the categorical variables and as position statistics (average, minimum, maximum) and standard deviations (SDs) for the continuous variables.

The average age of the patients was 11.8 (±6.4) years. The Kappa test demonstrated good interexaminer agreement for behavioral evaluation using the VSs (0.81), demonstrating a considerable decrease between the initial and subsequent assessments.

The patients required 10 to 60% N_2_O for sedation, average of 35.6% (SD ± 10.4) (Table 2).

Table 3 expresses the VS variation between the times, categorizing them as “no collaborator” and “collaborator” for treatment.

In Table 3, it is noted that the inhalation sedation was effective. Only 10.4% of patients in T1 allowed approach. Immediately after sedation (T2), 89.6% showed conditions of treatment (p < 0.001). From T2 to T4, McNemar test did not reject the null hypothesis that the situation of patients is maintained over time. The patient’s behavior remained stable until the end of the care (Table 3).

It is noted that the HR of those which did not allow the treatment remained elevated when starting the procedure, approximately 15 bpm higher than that ones who allowed the work. Although, even for that stratum, it was observed a decrease of the initial HR (Table 4).

In Table 5, comparisons between categorical variables of noncollaborator and collaborator groups according to VS and the continuous variable HR were adjusted for age. Table 5 interprets the percentage of individuals who cooperated or not with dental treatment.

**Table 2:** **Table 2:** Descriptive variables of ages, RR, sedation time, HR, N_2_O%, O_2_%, and VS, according to the study group

*Variable*		*n*		*Minimum*		*Maximum*		*Average*		*SD*		*Median*	
Age (years)		77		3		32		11.8		6.4		10	
RR T1 (bpm)		77		14		18		16.2		1.2		16	
RR T2 (bpm)		77		14		18		16.1		1.0		16	
RR T3 (bpm)		77		14		18		16.0		1.0		16	
RR T4 (bpm)		77		14		18		16.0		1.0		16	
Sedation time		77		10		90		34.4		14.9		30	
HR T1 (bpm)		77		70		198		115.7		21.0		113	
HR T2 (bpm)		77		55		130		83.4		16.6		80	
HR T3 (bpm)		77		60		159		85.0		18.9		80	
HR T4 (bpm)		77		57		140		87.5		19.8		89	
N_2_O (%)		77		10		60		35.6		10.4		40	
O2 (%)		77		40		95		64.6		10.8		60	
VS (T1)		77		1		5		4.2		0.8		4	
VS (T2)		77		0		5		1.8		1.3		1	
VS (T3)		77		0		5		1.9		1.4		2	
VS (T4)		77		0		5		1.8		1.4		2	

For continuous variables: T1: Presedation; T2: Optimal titration; T3: Sedated patient (right after the procedure); T4: Oxygenation (after removal of N_2_O); Venham’s results: 0: Relaxed and fully cooperative; 1: Apprehensive; 2: Tense; 3: Reluctant; 4: Interruption; and 5: Without communication

**Table 3:** **Table 3:** Percentage of patients who cooperated in the dental treatment, according to the VS, at times T1, T2, T3, and T4

*Time*				*Non collaborator*		*Collaborator*		*Total*		*p-value^a^*	
T1		N		69		8		77		–	
		%		89.6		10.4		100			
T2		N		8		69		77		<0.001	
		%		10.4		89.6		100			
T3		N		11		66		77		0.24	
		%		14.3		85.7		100			
T4		N		9		68		77		0.61	
		%		11.7		88.3		100			

T1: Presedation; T2: Optimal titration; T3: Sedated patient (right after the procedure); T4: Oxygenation (after removal of N_2_O); ^a^McNemar test in relation to previous moment

Regarding HR, the p values were borderline significant (0.06, 0.09, and 0.10 at times T2, T3, and T4 respectively). Table 4 demonstrates that this relationship was significant. Because the categorization of a measurement reduces the power, a greater number of patients are required to identify significant variations.

**Graph 1:**
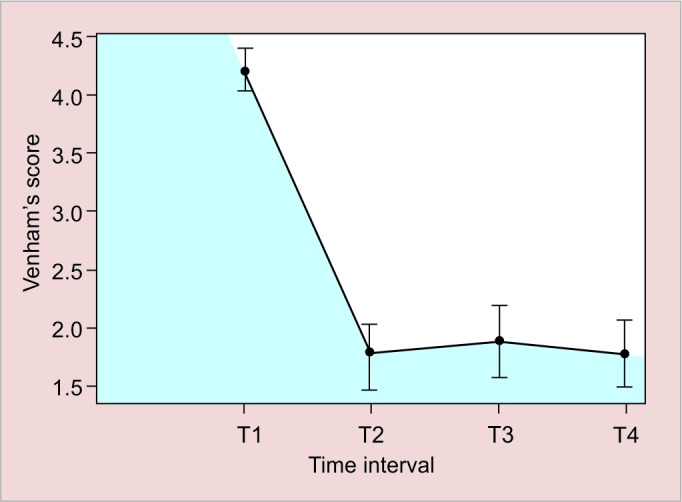
Respiratory rate average profile over time

Graph 1 and Table 6 tests the time evolution of response measures, assessing HR and RR variables in times T1, T2, T3, and T4. For each variable, the average and confidence intervals are 95%.

The general p-value of Brunner et al^21^ model for repeated measurements for RR over time was 0.12, indicating that there is no evidence that RR varies over time (Table 6).

**Graph 2:**
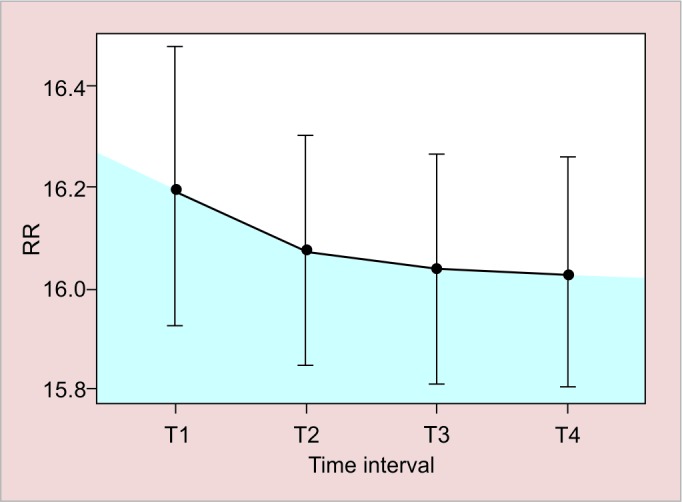
Heart rate average profile over time

Graph 2 shows the comparison for the HR variable between T1, T2, T3, and T4 times.

**Table 4:** **Table 4:** Comparison between means of variables of age, RR, HR, N_2_O%, and O_2_%, according to the categories of VS at T3

		*Noncollaborator (n = 11)*				*Collaborator (n = 66)*				*Total (n = 77)*					
*Variable*		*Mean*		*SD*		*Mean*		*SD*		*Mean*		*SD*		*p-value^a^*	
Age (years)		10.27		5.35		12.08		6.6		11.82		6.43		0.39	
RR T1 (bpm)		16.09		1.14		16.21		1.25		16.19		1.23		0.76	
RR T2 (bpm)		16		1		16.09		1.02		16.08		1.01		0.78	
RR T3 (bpm)		16.09		1.14		16.03		0.99		16.04		1.01		0.85	
RR T4 (bpm)		16		1		16.03		0.99		16.03		0.99		0.92	
HR T1 (bpm)		123.82		25.95		114.39		19.94		115.74		20.97		0.16	
HR T2 (bpm)		95.09		9.75		81.41		16.7		83.36		16.56		0.010	
HR T3 (bpm)		97.64		11.63		82.86		19.14		84.97		18.93		0.016	
HR T4 (bpm)		101.36		15.7		85.2		19.57		87.51		19.81		0.011	
N_2_O (%)		33.18		8.74		36.06		10.62		35.65		10.37		0.39	
O_2_ (%)		67.73		9.05		64.09		11.06		64.61		10.81		0.30	

^a^Student’s t-test

**Table 5:** **Table 5:** Comparison between means of categorical variables according to categories of VS after the procedure (T3)

				*Allows work (Venham T3 ≤ 3)*													
				*Non collaborator (n = 11)*				*Collaborator (n = 66)*				*Total (n = 77)*					
*Variable*		*Factor*		*n*		*%*		*n*		*%*		*n*		*%*		*p-value^a^*	
CP types		Choreoathetosis		1		25.0		3		75.0		4		100		0.19	
		Diparesis		4		16.7		20		83.3		24		100			
		Hemiparesis		1		3.6		27		96.4		28		100			
		Tetraparesis		5		23.8		16		76.2		21		100			
HR (T1)		Bradyc. for age		0		–		0		–		0		100		1	
		Normal for age		3		15.0		17		85.0		20		100			
		Tachyc. for age		8		14.0		49		86.0		57		100			
HR (T2)		Bradyc. for age		1		3.3		29		96.7		30		100		0.06	
		Normal for age		7		18.9		30		81.1		37		100			
		Tachyc. for age		3		30.0		7		70.0		10		100			
HR (T3)		Bradyc. for age		1		3.4		28		96.6		29		100		0.09	
		Normal for age		7		19.4		29		80.6		36		100			
		Tachyc. for age		3		25.0		9		75.0		12		100			
HR (T4)		Bradyc. for age		1		3.4		28		96.6		29		100		0.10	
		Normal for age		7		20.0		28		80.0		35		100			
		Tachyc. for age		3		23.1		10		76.9		13		100			

Tachyc.: Tachycardia; Bradyc.: Bradycardiac; ^a^Chi-square test

Heart rate showed a significant difference (p < 0.001), and therefore, multiple comparisons of nonparametric Wald model for HR measures repeated two by two were made. Multiple comparisons in Table 6 indicate a large reduction in HR from T1 to T2, which continues to decrease from T2 to T3 and stabilizes until T4. In the analysis of Table 2, we note that there is an increase in the HR average at T2 to T3; however, the nonparametric model assumes that there is a reduction in scale of measurement, because that increase in average is because of two patients (#45 and #54) having a nonstandard increase of HR (Table 6).

**Graph 3:**
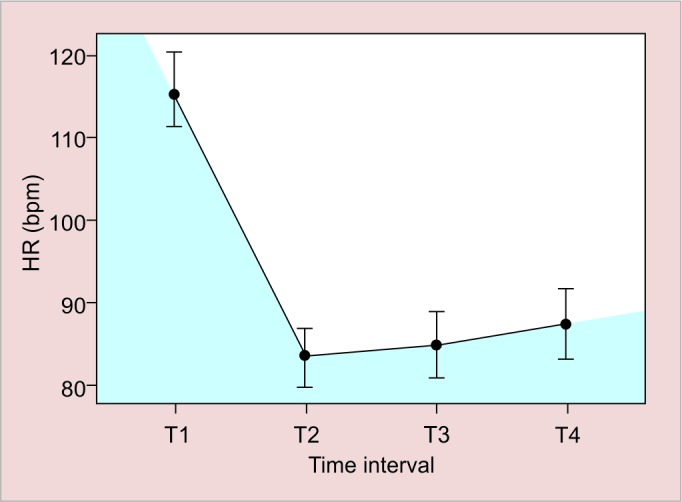
Venham score average profile over time

Graph 3 assessed the effect of the N_2_O sedation associated with VS along the times T1, T2, T3, and T4 and notes a significant drop in scores at T1 to T2, then a small increase from T2 to T3, which can be explained by the procedure itself and not by sedation. The increase observed between T2 and T3 did not have clinical relevance, because even with this alteration, patients remained collaborators in VS.

**Table 6:** **Table 6:** Multiple comparisons between times for HR variable

*Time interval*		*Statistics*		*Degrees of freedom*		*p-value*	
1-2		247.829		1		<0.001	
1-3		157.239		1		<0.001	
1-4		135.932		1		<0.001	
2-3		6.021		1		0.01	
2-4		9.006		1		0.002	
3-4		1.776		1		0.18	

**Table 7:** **Table 7:** Wald Multiple comparisons of nonparametric model for repeated measures for VS

*Time*		*Statistics*		*Degrees of freedom*		*p-value*	
1-2		251.631		1		<0.001	
1-3		204.057		1		<0.001	
1-4		225.862		1		<0.001	
2-3		4.769		1		0.02	
2-4		0.327		1		0.56	
3-4		3.141		_1_		0.07	

As in different times, there were significant differences; multiple comparisons were made two by two with nonparametric test to assess at what time there is an important difference (Table 7).

The percentage used for N_2_O is not influenced by the characteristics of the different subgroups studied.

## DISCUSSION

This was the first study to demonstrate the effects of N_2_O via evaluations of the physiological and behavioral parameters pertaining to patients with CP. The results obtained herein support the use of sedation with N_2_O to provide dental care to patients with CP. This discussion will focus on the primary parameters for this indication.

Patients with CP represent a group of individuals with heterogeneous clinical manifestations presenting with different types of movement disorders, such as spasticity, dyskinesia (athetosis/dystonia), and ataxia, as well as different types of clinical standards (tetraparesis, diparesis, hemiparesis). These individuals present with greater incidences of caries and periodontal disease.^23^ The patients assessed in the present study exhibited the characteristics described above, including choreoathetoid movements (5.19%), diparesis (31.16%), hemiparesis (36.36%), and tetraparesis (27.27%).

The profile of this study’s population demonstrated that most of the patients were between 3 and 32 years of age and exhibited similar clinical patterns, including tetraparesis, diparesis, and hemiparesis. Dyskinetic (choreoathetoid) patients represented 5% of the sample, which was a smaller proportion than that noted in previous studies involving patients with CP.^1^

N_2_O has a low anesthetic potency. Under normal temperature and pressure, a minimum alveolar concentration of 104% is required to achieve an anesthetic effect.^24^ Due to this pharmacodynamic feature, N_2_O is generally used in combination with either inhaled or IV anesthetics in the setting of general anesthesia.^24-26^ Therefore, under the conditions used in the present study, N_2_O induced only sedation, without the risks associated with general anesthesia. Due to the criticisms and warnings in the literature regarding the safety of using N_2_O in the setting of anesthesia, its use remains infrequent.^26^ The absence of clinical complications in the present trial reinforces the idea that N_2_O may be used as a resource to provide dental care to patients with special needs. An inorganic agent, N_2_O does not affect hepatic metabolism and is a safe option for sedation among patients, who require long-term medication.

The combination of N_2_O and psychoactive drugs typically potentiates its sedative effects.^27-29^ According to the data obtained herein, there was no potentiation of the sedative effects of N_2_O due to the concomitant use of psychoactive drugs. The RR was not altered throughout the entire procedure (Table 3). The Brondani et al^30^ data regarding the relationship between psychoactive drugs and N_2_O indicated that there were no alterations in the RR, a finding that supported the clinical safety of the concomitant use of N_2_O and psychoactive drugs among patients with CP.

The results obtained regarding the variation in the RR due to the use of N_2_O demonstrated no statistically significant alterations. As stated in the literature, N_2_O may selectively affect the cerebral cortex and, therefore, does not depress the respiratory centers located in the bulb.^11^

One of the most difficult aspects of dental treatment in patients with CP is related to their neurological motor disorders. Depending on the degree of these disorders, professional stabilization measures and patient restraints are often required to provide safer dental treatment.

In this study, 32.4% of the patients exhibited severely impaired mobility and required clinical restraints for stabilization. Sedation techniques that promote general muscle relaxation are fundamental to performing dental procedures. Therefore, although there was no correlation between severe mobility impairments and N_2_O titration, its use is recommended to facilitate patient cooperation during dental procedures as evidenced by the analysis of the VS variation, observed in Graph 3.

Autonomic dysfunction in patients with CP has been described by Yang et al.^31^ An imbalance in sympathovagal swing occurs in the autonomic nervous system, as evidenced by the high values obtained for HR, characterized by the predominance of the sympathetic nervous system, in patients with CP.^32^ Dental treatment generates fear and anxiety, which justifies the implementation of control via safer sedation methods. Knowledge regarding the effects of conscious sedation with N_2_O, including both inotropic and negative chronotropic effects, reduces the potential for cardiovascular complications among patients with CP.

In the present study, 32.8% of the patients with CP who were sedated with N_2_O developed bradycardia (p = 0.07). Although these findings were statistically significant, the bradycardia did not affect the patients’ physiological conditions from a clinical perspective because the patients’ HRs were maintained above 80 bpm, a finding indicative of a safe physiological pattern.^33^

An autonomic imbalance characterized by the predominance of sympathetic action is often present in patients with CP.^8^ The decrease in HR within physiological limits between T1 and T2 (p = 0.04) reinforced the validity of the indications for conscious sedation with N_2_O under these conditions.

Our data demonstrated the effectiveness of N_2_O in reducing HR and anxiety, as expressed using the VSs, confirming the findings presented in the literature.^30,34,35^

The parameters used to evaluate anxiety, which were expressed using the VSs, demonstrated that patients with CP exhibited a significant response to N_2_O. Our data demonstrated that 89.6% of the patients classified as noncollaborators exhibited significant reductions in their anxiety levels (p < 0.001), which were sufficient to reclassify them as collaborators. The patients were more cooperative and less agitated during the procedure, supporting the viability of the treatment to provide greater comfort and tranquility for the patient, their escorts, and the professional team.^35-38^

The highest frequency of patients who were noncolla-borators was noted among the patients with CP categorized as choreoathetoid and tetraparetic. This finding may be attributed to mechanical difficulties in maintaining the mask in the proper position due to the patients’ involuntary movements. Because they represented a small subgroup within the study population, we believe that additional studies including a larger number of patients are necessary to confirm this hypothesis.^39^

## CONCLUSION

The use of conscious sedation with N_2_O controls stress during dental care in individuals with CP. Sedation modifies behavior during dental procedures, facilitating patient collaboration. No respiratory depression or decreases in HR were observed in response to the use of conscious sedation during dental care.

## CLINICAL SIGNIFICANCE

The control of stress during the dental treatment using N_2_O reduces the fear and anxiety of CP patients and enhances their cooperation during dental procedures. Thus, the conscious sedation with N_2_O could be used as an effective and safety clinical technique to improve the dental care in patients with CP.
